# Induction of Atypical Autophagy by Porcine Hemagglutinating Encephalomyelitis Virus Contributes to Viral Replication

**DOI:** 10.3389/fcimb.2017.00056

**Published:** 2017-02-28

**Authors:** Ning Ding, Kui Zhao, Yungang Lan, Zi Li, Xiaoling Lv, Jingjing Su, Huijun Lu, Feng Gao, Wenqi He

**Affiliations:** ^1^Key Laboratory of Zoonosis, Ministry of Education, College of Veterinary Medicine, Jilin UniversityChangchun, China; ^2^Key Laboratory of Zoonosis, Ministry of Education, Institute of Zoonosis, Jilin UniversityChangchun, China

**Keywords:** porcine hemagglutinating encephalomyelitis virus, atypical autophagy, neurotropic virus, neuro-2a cells, virus replication, autophagic flux, LC3

## Abstract

Autophagy is a basic biological metabolic process involving in intracellular membrane transport pathways that recycle cellular components and eliminate intracellular microorganisms within the lysosome. Autophagy also plays an important part in virus infection and propagation. However, some pathogens, including viruses, have evolved unique trick to escape or exploit autophagy. This study explores the mechanism of autophagy induction by porcine hemagglutinating encephalomyelitis virus (PHEV) in Neuro-2a cells, and examines the role of autophagy in PHEV replication. PHEV triggered autophagy in Neuro-2a cells is dependent on the presence of bulk double- or single-membrane vacuoles, the accumulation of GFP-LC3 fluorescent dots, and the LC3 lipidation. In addition, PHEV induced an incomplete autophagic effect because the degradation level of p62 did not change in PHEV-infected cells. Further validation was captured using LysoTracker and lysosome-associated membrane protein by indirect immunofluorescence labeling in PHEV-infected cells. We also investigated the change in viral replication by pharmacological experiments with the autophagy inducer rapamycin or the autophagy inhibitor 3-MA, and the lysosomal inhibitor chloroquine (CQ). Suppression of autophagy by 3-MA increased viral replication, compared with the mock treatment, while promoting of autophagy by rapamycin reduced PHEV replication. CQ treatment enhanced the LC3 lipidation in PHEV-infected Neuro-2a cells but lowered PHEV replication. These results show that PHEV infection induces atypical autophagy and causes the appearance of autophagosomes but blocks the fusion with lysosomes, which is necessary for the replication of PHEV in nerve cells.

## Introduction

Porcine hemagglutinating encephalomyelitis virus (PHEV) is a single-stranded, positive-sense RNA coronavirus and is classified as a member of the *Coronaviridae* family (Andries and Pensaert, 1981). It mainly gives rise to encephalomyelitis or vomiting and wasting disease in piglets. As a neurotropic virus, it spreads via the peripheral to the central nervous system (CNS), resulting in neurological damage by virus infection (Andries and Pensaert, 1980). Nervous lesions caused by pathogens often lead to abnormal nerve cell morphology, unnatural protein accumulation, vesicular transport or neuronal gene transcription disorders, and synaptic transmission obstacles (Xilouri and Stefanis, 2010). Similarly, as a virus invading the nervous system, rabies virus (RABV) can cause severe encephalitis and induce analogous cytopathic features (Peng et al., 2016). Although, some studies of PHEV pathogenesis have been performed, the underlying mechanism of PHEV replication is still poorly elucidated. Therefore, a more detailed description of the mechanism should be revealed, especially the biological changes of the host cells induced by infection.

In eukaryotic cells, autophagy is characterized by its highly conserved as a metabolic process for preserving the homeostasis. Among the intracellular membrane transport pathway, the corresponding cytoplasmic proteins and cargos are delivered to the lysosomes (Levine et al., 2011). As a cytoprotective process, autophagy is an inherent host defense mechanism against viral aggression, which helps to maintain cell homeostasis in response to multiple external stress stimuli, containing a nutrient-poor environment, endoplasmic reticulum (ER) stress, especially pathogen infection (Kroemer et al., 2010). Dysfunction in autophagy has been associated with many illnesses that infect humans such as neurodegeneration diseases, cancer, metabolic syndrome, lysosomal diseases, and aging (Rubinsztein et al., 2011).

Previous studies have indicated that autophagy not only protects cells under stressful conditions, but also plays an important part in the process of pathogen infection (Shintani and Klionsky, 2004). Some viruses have also developed strategies to reverse the autophagy defense pathway. US11, a late gene of herpes simplex virus-1 (HSV-1), blocks the formation and development of autophagosomes in both fibroblasts and HeLa cells. US11 also interacts with PKR to suppress autophagy (Lussignol et al., 2013). Nevertheless, there are viruses that utilize autophagosomes for promoting their replication, such as in poliovirus infection, where the formation of autophagosomes provides a conservative platform for viral replication (Suhy et al., 2000). Conversely, in the Sindbis virus and tobacco mosaic virus infections, autophagy successfully suppresses viral replication (Liu et al., 2005). More interestingly, several coronaviruses can hijack the important autophagy-related protein LC3-I and endoplasmic reticulum vesicle structures for their replication (Reggiori et al., 2010) using the same mechanism as rotavirus (Crawford et al., 2012). This mechanism induced by viral proteins is associated with their own pathogenic non-structural proteins, exemplified by the infectious bursal disease virus infection (Hu et al., 2015). However, whether PHEV, as a member of the coronavirus family, infection is associated with autophagy is unknown. In this study, we confirmed that PHEV infection induces atypical autophagy and leads to the accumulation of autophagosomes while blocking their fusion with lysosomes, which creates conditions for the virus to replicate within nerve cells.

## Materials and methods

### Cells, viruses, and plasmids

Mouse neuroblastoma (Neuro-2a) cells were cultured in high glucose Dulbecco's-modified Eagle's medium (DMEM) (GIBCO, USA) containing 2 mM L-glutamine and 1.5 g/L sodium bicarbonate, added with 10% fetal bovine serum (FBS). The PHEV strain HEV 67N (GenBank: AY048917) was propagated in N2a cells. The plasmid GFP-LC3 and the tandem fluorescent monomeric red fluorescent protein (mRFP)-GFP-LC3 (ptfLC3) were maintained in the laboratory.

### Viral infection, drug treatments, and cell viability assay

Neuro-2a cells were infected with PHEV (10^5.43^TCID_50_/mL) in cell cultures with 2% FBS for 1 h. Following a 1 h absorption period, infected cells were incubated in the complete DMEM at 37°C for the relevant times in the conformity with experimental requirements.

In subsequent experiments, the optimum concentration of the corresponding drug was used and the cell viability were determined by a WST-8 cell proliferation assay (Beyotime, China) according to the manufacturer's guidelines. The concentrations tested for rapamycin (Selleckchem, USA) were 100, 500, and 200 μM; for chloroquine (CQ) (Sigma, St. Louis, MO, USA) were 16, 32, 64, and 128 μM; and for 3-methyladenine (3-MA) (Selleckchem, USA) were 1, 2, 3, and 5 mM. According to existing experimental procedures, the medium was removed with 100 μl of fresh medium added with 10 μl of WST-8, the sample was further incubated at 37°C for 1 h (Shao et al., 2014). Cell viability was evaluated by measuring the absorbance at 450 nm against the background control.

### Transmission electron microscopy (TEM)

TEM is a valid and important method for monitoring autophagy induction in morphology (Peng et al., 2016). In this assay, Neuro-2a cells were treated with 100 nM rapamycin in complete medium for 3 h as a positive control, and the infected cells were incubation with PHEV (10^5.43^TCID_50_/mL) for 24 h. The cell samples were washed three times with PBS, collected in the bottom of 1.5-ml Eppendorf tubes and centrifuged at 1,000 × g for 10 min. The cell pellets were fixed with 2.5% glutaraldehyde in PBS overnight at 4°C and then postfixed in 1% OsO_4_ for 2 h. After being washed, the cells were dehydrated with a graded series of ethanol and then embedded in epoxy resin. Next, ultrathin sections were prepared and stained with uranyl acetate and lead citrate as previously described (Risco et al., 2012). At last, the autophagosome-like vesicles were examined under a transmission electron microscopy (JEOL, Tokyo, Japan).

### Confocal fluorescence microscopy

Confocal fluorescence microscopy was utilized to detect the expression of LC3 in PHEV infected or drug-treated cells and the expression of autophagy-related proteins during PHEV infection. Neuro-2a cells were seeded in 22.1 mm dishes with coverslips and transfected with GFP-LC3, mRFP-GFP-LC3, or control plasmids. At 24 h post-transfection, the cells were incubated with complete medium with 10% FBS, and then mock-infected, and those infected with PHEV were treated as required. The cells were then incubated with 50 nM LysoTracker Red DND-99 (Invitrogen, Carlsbad, CA, USA) for 1 h, rinsed 3 times with PBS and analyzed under the Olympus FV1000 confocal microscope (Olympus, Japan).

### Western blotting analysis

Drug treated, mock-infected or PHEV-infected cells were harvested at corresponding times. Next, immediately lysed with RIPA lysis buffer, including 1 mM PMSF (Beyotime, China) for 30 min to 1 h on ice, shaking during the lysis. The clarified lysate was boiled in loading buffer for 10 min, and the proteins which in equal amounts were displayed on SDS–polyacrylamide gel electrophoresis (Millipore, Billerica, MA, USA). Afterwards, the proteins were transferred to PVDF membranes, blocked in PBST buffer added 5% nonfat milk powder for 1 h at 37°C. The corresponding primary antibodies were incubated overnight at 4°C, and the HRP-conjugated secondary antibodies for 1 h at 37°C.

### Autophagic flux measurements

p62, also called SQSTM1, undergoes degradation in the course of autophagy. Whether the complete autophagy response was activated by PHEV infection, we analyzed p62 degradation by western blotting or confocal immunofluorescence microscopy for the autophagic flux as previously described (Bjørkøy et al., 2005). In this test, Neuro-2a cells were infected with PHEV and harvested at 6–48 h post-infection (hpi) using anti-p62 antibody as the primary antibody. CQ can inhibit autophagy by inhibiting acidification of lysosomes and endosomes. To verify the effect of PHEV infection on autophagic flux, the cells were infected with PHEV after CQ treatment, the levels of p62 and PHEV were detected.

A tandem reporter construct, mRFP-GFP-LC3 (ptfLC3), was used as an additional plasmid to monitor the autophagic flux (Kimura et al., 2007). Neuro-2a cells grown to 60–70% confluency on coverslips were transfected with ptfLC3 and infected with PHEV (10^5.43^TCID_50_/mL). At 12, 24 and 48 hpi, the cells were fixed and visualized by confocal microscopy. GFP-LC3 colocalization with the lysosome-associated membrane protein 1 (LAMP1) was also investigated as d previously described. Briefly, Neuro-2a cells were transfected on cell slips with GFP-LC3 and infected with PHEV at 12, 24, and 48 h. Then, the cells were incubated and examined with rabbit antibody against LAMP1 (Abcam, Cambridge, MA, USA) under a confocal fluorescence microscope.

To mark the acidic late endosomal and lysosomal structures, LysoTracker Red (50 nM) was added to the treated, mock- or infected cells for 1 h. Following treatment or infection, cells were settled in 4% paraformaldehyde for 15 min and washed with PBS for three times, and then permeabilized using 0.1% Triton X-100 for 15 min at room temperature. Blocking was performed with 5% nonfat milk powder in PBS for 1 h prior to incubation with primary antibodies followed by incubation with the suitable secondary antibody. Images were visualized by confocal fluorescence microscopy.

### Quantitative real-time (qRT) PCR

Real-time quantitative reverse transcriptase polymerase chain reaction (qRT-PCR) was performed to analysis the virus copies of PHEV. The cells were given the appropriate treatment or infection and RNA was extracted following lysis in Tripure isolation reagent (Roche, Basel, Switzerland). According to the manufacturer's protocol of the PrimeScript® RT reagent Kit (Takara, Japan), the corresponding treated cDNA samples were synthesized. A pair of specific primers (PHEV-p1: AGCGATGAGGCTATTCCGACTA /PHEV-p2: TTGCCAGAATTGGCTCTACTACG) was used in the study for PHEV detection, which was targeting a region to the HE gene of PHEV. The real-time RT-PCR was performed using FastStart Universal SYBR Green Master (Roche). The PCR conditions were as follows: 95°C for 3 min (1 cycle), 95°C for 30 s, 60°C for 30 s, and 72°C for 30 min (40 cycles).

## Results

### Autophagosomes accumulate in PHEV-infected cells

**Figure 1:**
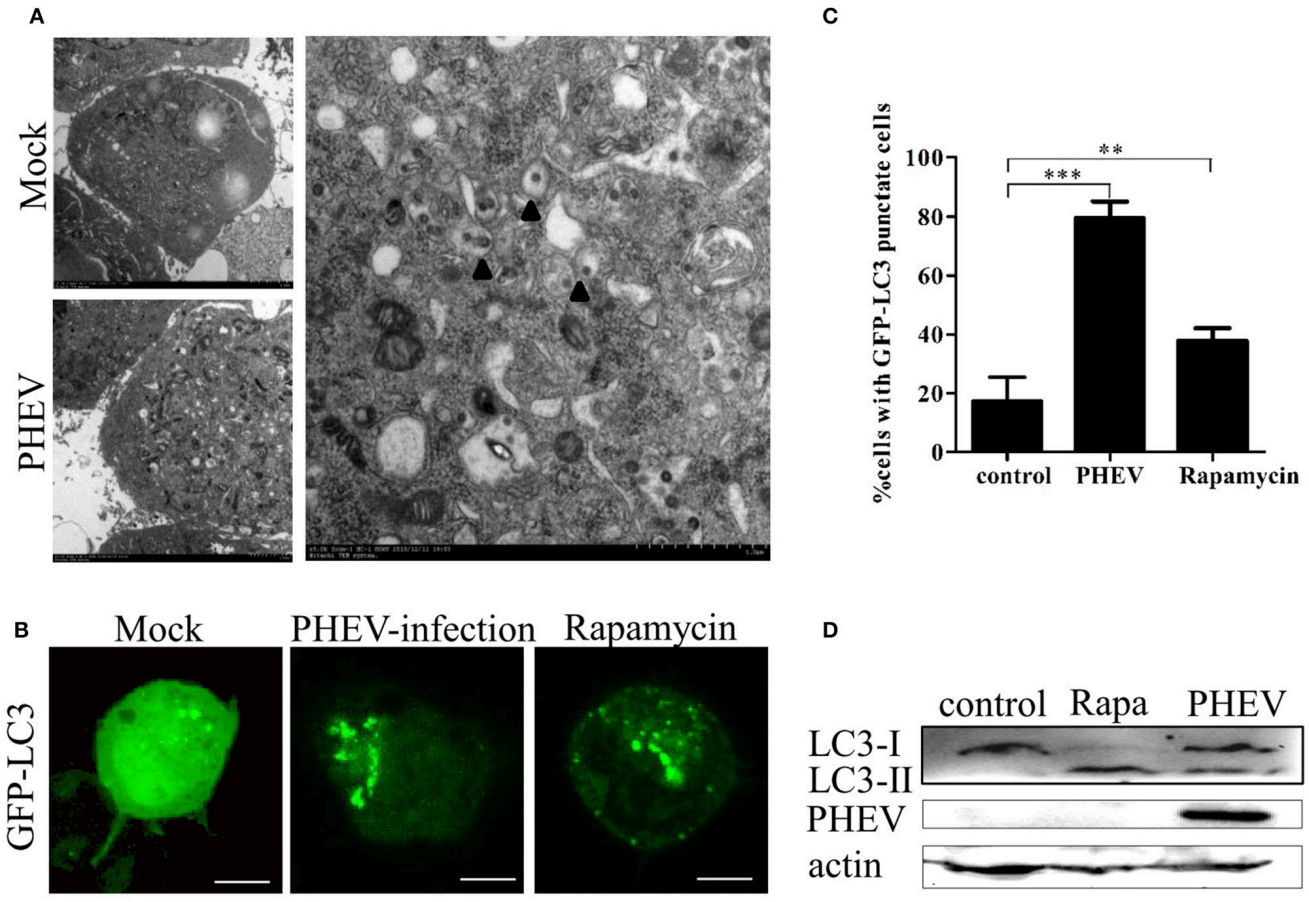
**Autophagosomes accumulate in PHEV-infected Neuro-2a cells. (A)** TEM observations. Neuro-2a cells were mock infected or infected with PHEV for 24 h and studied by transmission electron microscopy. Black triangles indicate the structures with autophagosomes characteristics. **(B)** Confocal microscopy. Neuro-2a cells were transfected with GFP-LC3 followed by treatment at 24 h post transfection with mock treatment as a negative control, and rapamycin treatment as a positive control. The localization of GFP-LC3 positive autophagosome accumulation (green) and the S-tagged PHEV products (red) was visualized using a confocal microscope. **(C)** The quantification of cells showing GFP-LC3 puncta in PHEV-infected cells. In three random fields, the average number of puncta in each cell was taken from at least 80 cells in each treatment. Representative results with graphs are shown in Figure 1B. **(D)** Western blotting. The turnovers of LC3-I to LC3-II were detected for mock-treated Neuro-2a cells, rapamycin-treated Neuro-2a cells, and PHEV-infected Neuro-2a cells. Cells were collected at appropriate time points and detected with anti-LC3B antibody, and β-actin was used as a protein loading control. ^**^P < 0.01; ^***^P < 0.001. Scale bars: 10 μm.

In this study, double membrane vacuoles containing organelle and cytosolic components, which are analogous to autophagosomes, were visible in the PHEV infected cells compared with the mock cells (Figure 1A). To understand how PHEV induces autophagy, we monitored the processing of LC3-I to its lipidated membrane-bound form LC3-II. The Neuro-2a cells were transfected with GFP-LC3 and prepared for rapamycin for 6 h or PHEV-infection (Figure 1B). In the mock-infected Neuro-2a cells, GFP-LC3 shows a diffuse distribution (Figure 1B). PHEV infection leads to the accumulation of autophagosomes in cells, as observed by GFP-LC3 punctate redistribution. Similarly, short-term treatment with rapamycin efficiently induced autophagy, resulting in GFP-LC3 puncta formation in ~40% of cells (Figure 1C). To further corroborate the finding that PHEV induces autophagy, we measured the expression of LC3-I and LC3-II in cells by western blotting. Consistent with the GFP-LC3 fluorescence and TEM data, an increase of LC3-II was detected in the rapamycin treated and PHEV-infected cells (Figure 1D).

### The autophagy by PHEV-induced is time-dependent

**Figure 2:**
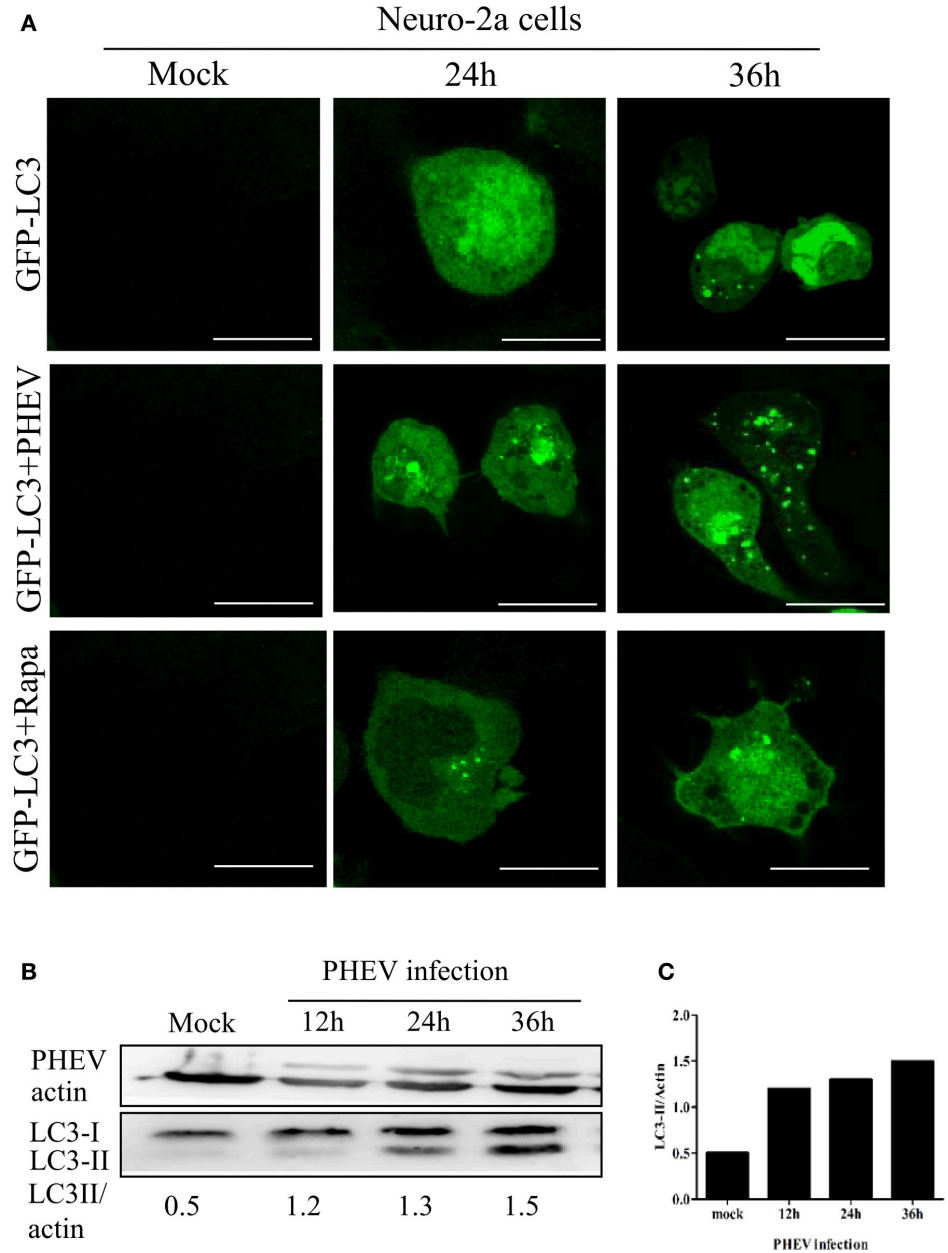
**PHEV-induced autophagy was time-dependent. (A)** Immunofluorescence microscopy was used to detect the GFP-LC3-autophagosomes in PHEV infected cells at different post-infection time points before transfection with GFP-LC3 into Neuro-2a cells. The cells were fixed at 0, 24, and 36 h post-infection, respectively, and then analyzed for GFP-LC3 positive autophagosome accumulation using a confocal microscope as described in Figure 2A. **(B,C)** Neuro-2a cells were infected with PHEV and mock-infected cells served as control. At 0, 12, 24, and 36 h post-infection, the cells were then lysed for western blotting analysis using an anti-LC3B and anti-β-actin antibodies. Scale bars: 10 μm.

To investigate the temporal regulation of autophagy by PHEV, a time course of PHEV infection showed that autophagosome accumulation could be detected by Western blotting from 6 to 36 hpi, and it increased with the infection process (Figure 2A). In addition, immunofluorescence microscopy was used to detect the GFP-LC3 puncta and contrast with the cells transfected with control vector showing that, GFP-LC3 puncta steadily increased at 36 hpi (Figure 2B). In line with these data, increased LC3-II accumulation was also examined in PHEV-infected cells with the increasing time by infection (Figure 2C).

### The membranes of autophagosome-like vesicles have colocalization with virions

**Figure 3:**
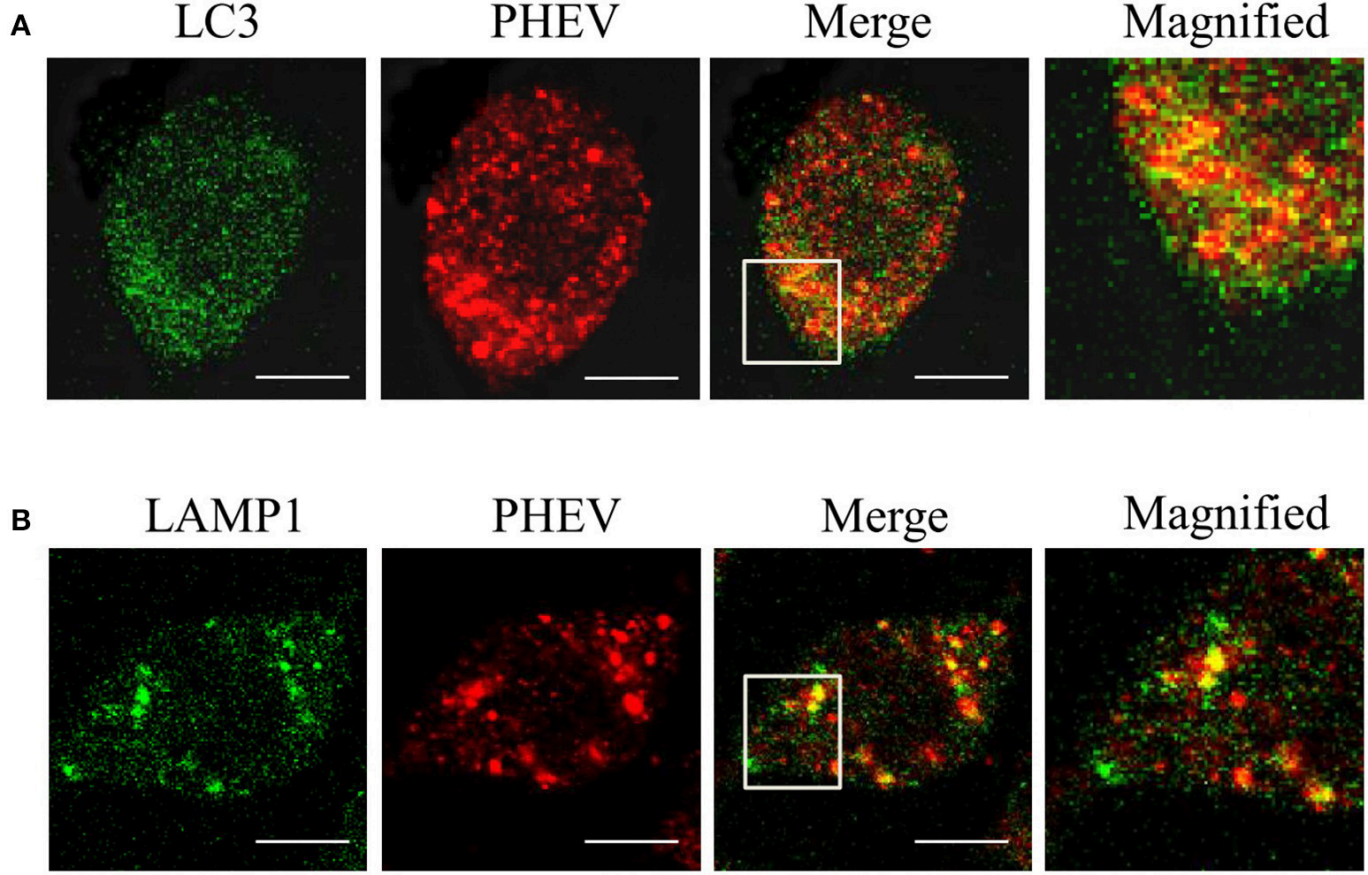
**The membranes of autophagosome-like vesicles have colocalization with virions. (A,B)** PHEV-infected Neuro2a cells were stained with LC3 (green) and PHEV (red) antibodies. The color-merged images are shown in the third panel. The colocalization between PHEV and LC3 and LAMP1 was magnified in the merged images. Scale bars: 10 μm.

In order to examine whether the autophagy is associated with viral replication in PHEV infection, we performed subcellular localization of viral proteins and LC3 or LAMP on PHEV-infected cells for the first time. As shown in the Figures 3 A,B, the effective fluorescent signal is observed in the infected cells, with puncta accumulation. In addition, we also examined the fluorescence spectra of LAMP1 in PHEV-infected cells. We found that LAMP1 can is localized with the viral protein in the infected cells. It reveals that the reassignment of autophagy marker LC3 induced by PHEV appeared in the infected cells and the autophagy is involved in viral replication.

### PHEV infection suppresses autophagic flux

**Figure 4:**
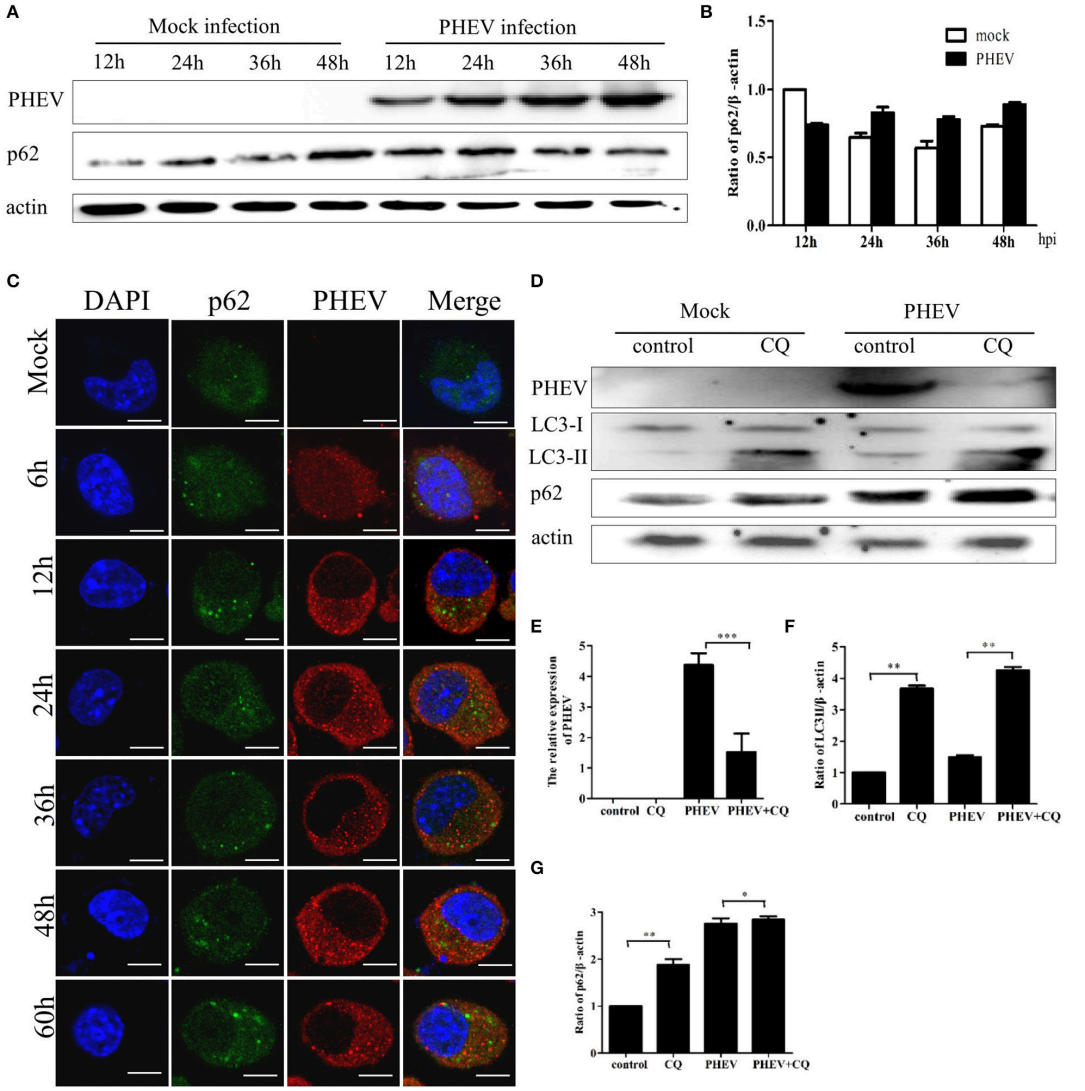
**PHEV infection suppresses autophagic flux. (A)** Western blot analysis of p62 changes in Neuro-2a cells with PHEV infection for 12, 24, 36, and 48 h, as well as the mock infection for the relevant times. **(B)** The p62 to β-actin ratio normalized to the mock infection set at 1.0 of **(A)** (*n* = 3; *P* < 0.05). **(C)** Representative images of Neuro-2a cells with PHEV infection from 6 to 60 h labeled with antibodies to anti-p62 and anti-PHEV. **(D)** Effect of chloroquine (CQ) treatment on LC3-II and p62 of Neuro-2a cells for 24 h, as tested by Western blotting. **(E–G)** show the ratios of PHEV to β-actin, p62 to β-actin or LC3-II to β-actin from three independent experiments of **(D)**. ^*^P < 0.05; ^**^P < 0.01; ^***^P < 0.001. Scale bars: 10 μm.

The complete autophagy process contains the formation of autophagosomes and the fusion between autophagosomes and lysosomes (Kliosnky et al., 2016). Immunoblotting showed that PHEV-infected cells have no significant changes in the p62 protein from 12 to 48 hpi, compared to the mock infection (Figures 4A,B). Consistent with these results, the levels of the p62 protein were essentially invariant during the infection by PHEV determined by confocal fluorescence microscopy (Figure 4C).

Furthermore, the cells were infected with PHEV after CQ treatment, the result showed that the obvious higher accumulation levels of p62 than that of the control (Figure 4D). As shown in Figures 4E–G, CQ increased the levels of LC3II and p62 and suppressed PHEV infection. These results suggest that cells infected with PHEV suppress autophagic flux, leading to no decrease of p62 but an accumulation when treated with CQ before PHEV infection. These results also revealed that PHEV induced incomplete autophagy. These data implied that PHEV infection may interfere with the fusion of autophagosomes with lysosomes or impair autolysosomal degradation.

### PHEV activates autophagosome formation but suppresses its fusion with lysosomes

**Figure 5:**
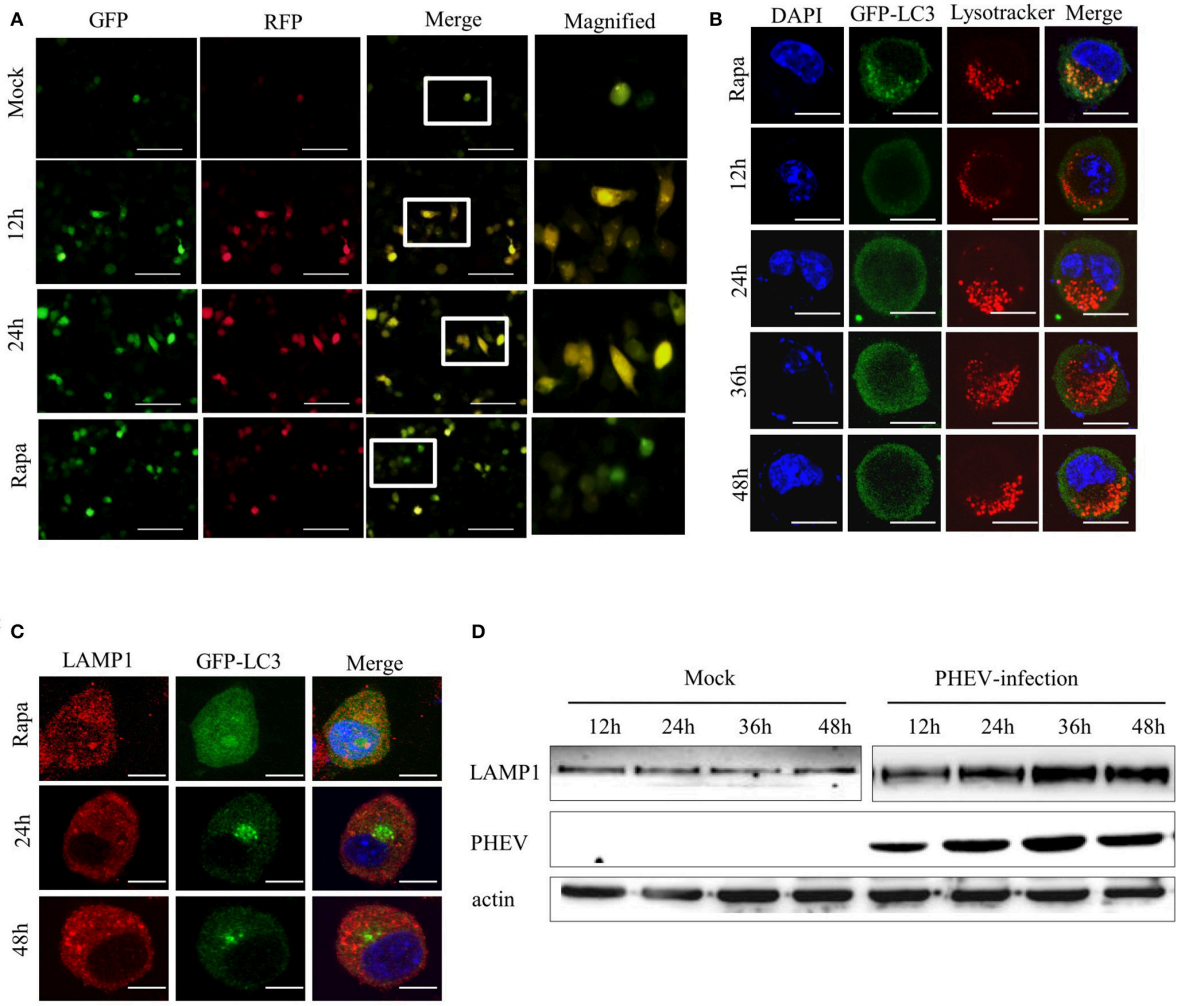
**PHEV activates autophagosome formation but prevents its fusion with lysosomes. (A)** Neuro-2a cells were transfected with mRFP-GFP-LC3. As the positive control for induction of autophagy, Neuro-2a cells were transfected with mRFP-GFP-LC3 and then treated with complete medium supplemented with 200 μM rapamycin for 3 h. At 0, 12, and 24 h post-transfection, the cells were fixed and assessed with GFP and mRFP fluorescence. Scale bars: 50 μm. **(B)** Neuro-2a cells were used to analyze the colocalization of LysoTracker-stained acidified vesicles and GFP-LC3-positive autophagosomes in the mock-infected and PHEV-infected cells at 12, 24, 36, and 48 h. Representative images are shown in Figure 5B. **(C)** Furthermore, the fusion of autophagosomes with lysosomes was analyzed as colocalization of the autophagosome marker GFP-LC3 with the lysosome marker LAMP1. Nuclear DNA was stained with DAPI. One of the three experiments conducted is shown. **(D)** Western blotting analysis of LAMP1 changes in PHEV-infected cells for 12, 24, 36, and 48 h were shown in Figure 5D, compared with the relevant times in mock cells. Scale bars: 10 μm.

According to the results presented above, PHEV infection induces an autophagic response, but efficient autophagic protein degradation and the complete autophagic process remains unobserved. In order to further explore the potential mystery, which of the inhibition of autophagic degradation induced by PHEV, we transfected the Neuro-2a cells with a tandem-tagged fluorescent reporter, mRFP-GFP-LC3 (ptfLC3) (Figure 5A). The GFP fluorescence from the ptfLC3 fusion protein is quenched in acidic autolysosomal conditions; however, the red fluorescence signal produced by ptfLC3 is not sensitive to acidic conditions. We found the GFP puncta were increased by PHEV during infection, as shown in Figure 5A. When both RFP and GFP signals were colocalized, resulting in yellow puncta, the RFP puncta remained increased at 12 and 24 h. However, with rapamycin treated cells, ptfLC3-transfected cells experienced complete autophagic flux and showed main red fluorescence with only a fuzzy green fluorescence.

Subsequently, we examined whether the fusion of the autolysosome with lysosomes was intact during PHEV infection. A marker for acidic late endosomal and lysosomal structures, LysoTracker Red was used in the corresponding treated cells (Figure 5B). In mock-infected cells, a certain number of GFP-LC3 vacuoles merged with LysoTracker staining. It indicated that a portion of autophagosomes had fused with lysosomes and became acidified. However, after PHEV infection, the autophagosomes do not fuse with acidic compartments.

To exclude the autophagosomes fusion with lysosomes rather than the efficiently acidified in infected cells, we also stained the cells with an antibody for LAMP1, a lysosomal marker. In accordance with the results above, the colocalization of GFP-LC3 and LAMP1 was not examined in Neuro-2a cells (Figure 5C). It was further clarified by western blotting, compared with the mock samples at the same time, that LAMP1 showed exceptional accumulation (Figure 5D). These data indicated that PHEV infection inhibits the fusion of autophagosomes with acidified LAMP1 lysosomes and thereby prevents the degradation of macroautophagy substrates. Collectively, these findings confirm that PHEV can induce autophagosome formation and activate an incomplete autophagic response.

### Induction of autophagy with rapamycin inhibits the replication of PHEV in Neuro-2a cells

**Figure 6:**
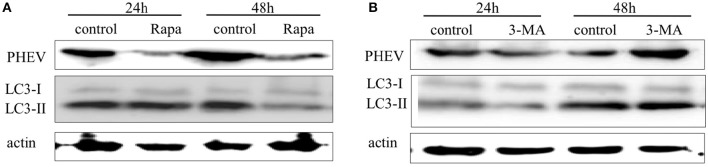
**The effect of -induced or -inhibited autophagy on PHEV replication. (A,B)** Neuro-2a cells were infected with PHEV in the presence or absence of rapamycin (100 nM) or 3-MA (2 mM). At 24 h and 48 hpi, the cells were harvested and analyzed by immunoblotting using anti-LC3, anti-PHEV, and anti-β-actin antibodies.

Autophagy may facilitate or suppress viral replication, depending on the virus type. To investigate the possible effect of autophagy on PHEV replication, we treated Neuro-2a cells with rapamycin, a prompter of autophagy that can stimulate complete autophagy, targeting mammalian target of rapamycin (mTOR) signaling pathways. We found that the induction of autophagy with rapamycin (200 μM) for 2 h effectively upregulated the alteration of LC3-I to LC3-II but significantly inhibited PHEV replication at 24 and 48 hpi in Neuro-2a cells (Figure 6A). It indicated that the complete autophagy pathway was activated after treatment with rapamycin; although, PHEV infection blocked the smooth of autophagic flow, PHEV replication was also inhibited because of the rapamycin treatment.

### Inhibition of autophagy with 3-methyladenine (3-MA) promotes the viral replication

In expect to recognize the effect of autophagy induced by PHEV infection on viral replication, we treated the Neuro-2a cells with 3-MA, the autophagic inhibitor targeting a class III phosphatidylinositol-3-kinase (PI3K) (Petiot et al., 2000). As shown in Figure 6B, there was no significant change in the conversion of LC3-I to LC3-II and the replication of PHEV at 24 hpi after 3-MA treatment. However, the replication of PHEV was significantly increased at 48 hpi after 3-MA treatment. These results suggested that 3-MA can promote effective PHEV replication by inhibiting autophagy.

### Regulation of autophagy does not affect cell viability

**Figure 7:**
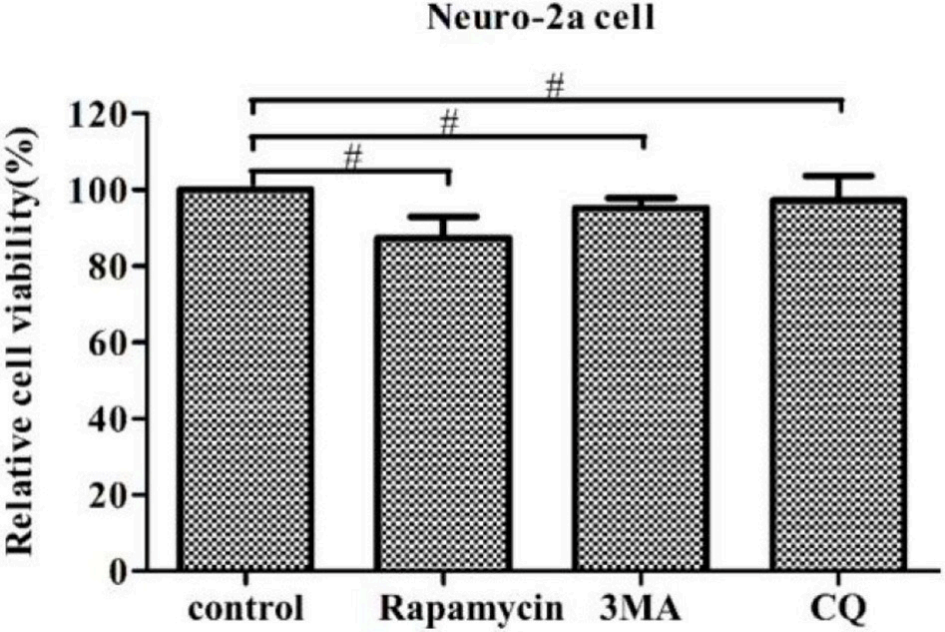
**Regulation of autophagy does not affect cell viability**. The pharmacological alteration of autophagy does not affect cell viability. Neuro-2a cells were determined by WST-8 cell proliferation assay after being treated with rapamycin, 3-MA, or chloroquine (CQ) for 24 h. Data on the percentage of cell viability are shown as the mean ± SD for three independent experiments. ^#^*P* > 0.05.

In this study, we examined the influence of autophagy during PHEV infection by pharmacological alterations of autophagy, containing rapamycin treatment, 3-MA treatment, and CQ treatment. We found that the cell viability was not significant change by WST-8 assay, which provided the basis for our further explore aiming to the relationship among autophagy and viruses (Figure 7).

## Discussion

PHEV is a neurotropic virus that transmits along the peripheral nerves to the CNS (Li et al., 2013). The neurological dysfunction by PHEV infection, results in neurological symptoms, vomiting and other clinical features. Some cases also accompanied with the characteristics of diarrhea, but whether the infection is associated with nerve injury is not yet clear (Li et al., 2016). In a previous study, we found that the invasion of nerve cells by PHEV is mediated by the S protein and neural cell adhesion molecule (NCAM), which is an adhesion molecule on the nerve cell surface (Gao et al., 2010; Dong et al., 2015). However, the specific mechanism by which the virus invades and replicates in nerve cells remains ill-defined. Autophagy plays a vital role in the replication of DNA and RNA viruses (Dreux and Chisari, 2010). Many RNA viruses induce autophagy in order to produce the membrane-associated replication complexes (Miller and Krijnse-Locker, 2008). During the coronavirus infection, LC3 could be recruited to form double-membrane vesicles (DMVs) from the ER (Reggiori et al., 2010). These DMVs appear to have a subcellular structure similar to the autophagosome, which is called a “virus factories” in a recent study. These viral factories provide a suitable platform for the replication and encourage autophagy, implying that a covered connection between DMVs and autophagy (Blanchard and Roingeard, 2015). In this study, it is an initial glance that PHEV infection induces atypical autophagy which is involved in viral replication.

Here, the traditional TEM assay were performed, the effectively increased number of double- or single-membrane vacuoles were displayed in PHEV infected cells for the first time, that is, the formation of autophagosome. We found that these single or double vesicle structure is similar to autophagosomes, but not exactly the same (Pei et al., 2014). Some of these vesicles encapsulate viral particles, while others do not. It suggested that autophagy induced by PHEV is different from the complete autophagy in conventional sense. In addition, accumulation of GFP-LC3 puncta in PHEV-infected cells were observed by laser confocal microscopy. It further verified that the formation of autophagosomes.

Autophagy is often closely related to viral replication, and several viruses use autophagic substances or the autophagosome to escape the immune system in order to achieve viral proliferation, while others use the autophagosome or autophagic vesicle model structure as its own site for genome transcription and replication (Jackson et al., 2005). Therefore, the development of autophagy often contributs to the proliferation of the virus (Levine and Kroemer, 2008). Several studies have determined the mechanism of virus-induced autophagy, such as influenza A virus. The virus inhibits the autophagic degradation process, and its matrix protein 2 plays a key role in this process (Gannagé et al., 2009). Rotavirus also blocks the maturation of autophagosomes and inhibits autophagy maturation, both of which may offer the rotavirus as a way for escaping the antiviral function of autophagy (Crawford et al., 2012). Recently, it has been reported that coronavirus membrane-associated papain-like protease PLP2 (PLP2-TM) could promote autophagy. In addition, the interaction between PLP2-TM with Beclin 1 affected the replication of the coronavirus, and further regulated the innate immune defense mechanism against viruses. (Chen et al., 2014). The similar phenomenon is shown in our results which demonstrated that PHEV could activate the appearance of autophagosome but influence its fusion with lysosomes.

As previously mentioned, the complete autophagy process involves the appearance of autophagosomes and the fusion and degradation of autophagosomes with lysosomes. The term “autophagic flux” is used to denote the dynamic process of autophagosome synthesis, the delivery of autophagic cargoes to the lysosome, and the degradation of autophagic cargoes inside the lysosome and is a more reliable indicator of autophagic activity than measurements of autophagosome numbers. Some recent studies have demonstrated that the induction of autophagy not only increases autophagosome formation and expression of autophagy proteins but also increases autophagic flux, which can be measured by detecting the levels of p62 (Pankiv et al., 2007). Using several means of measuring autophagic flux, including p62 degradation, LC3-II turnover, and GFP-LC3 lysosomal delivery and proteolysis, we confirmed that autophagic flux remained unchanged upon PHEV infection. The cells were infected with PHEV after CQ treatment, the level of LC3-II was increased, but PHEV infection was suppressed. The effect of CQ treatment may be related to its inhibition of autophagic proteolysis by suppressing acidification of lysosomes and endosomes. Cellular entry of coronaviruses is dependent on clathrin-mediated endocytosis, CQ treatment may block viral genome release from the lysosome into the cytoplasm for replication (Burkard et al., 2014). This may also be another dominant effect of CQ. These findings illustrate that an incomplete autophagy is activated in Neuro-2a cells by PHEV infected, like we mentioned as “atypical autophagy” above, and this autophagy induced by PHEV is involved in viral replication.

In some positive-strand RNA viruses, there is a co-localization between their viral components and autophagic markers, which subvert the autophagy mechanism, suggesting that the assembly of the translation/replication complexes of these viruses may exist in autophagic vesicles (e.g., poliovirus, mouse hepatitis virus, and dengue virus; Suhy et al., 2000; Lee et al., 2008; Beachboard et al., 2015). In the present study, we examined the co-localization between autophagy-related proteins and PHEV virion in infected cells, including LC3, an important component in the autophagosomal membrane; LAMP1, a widely used of lysosomal marker protein. We found that PHEV improved the rearrangement of LC3 and LAMP1, suggesting that several autophagy-associated membrane proteins are related to the PHEV replication.

In order to make a thorough inquiry the effect of autophagy on PHEV infection, Neuro-2a cells were treated with the corresponding experimental requirements in pharmacological alterations, including autophagy inducer (Rapa), the early autophagy inhibitor 3-MA and the late autophagy inhibitor CQ. Our data displayed that after rapamycin treatment, viral replication was inhibited by the rapamycin-treated autophagic effect. The early autophagic inhibitor 3-MA treatment could significantly promote the replication of PHEV, further implied that the formation of early autophagosome contributes to PHEV replication. At the same time, the results from the WST-8 assay we performed above proposed that the pharmacological alteration did not affect cell viability.

In conclusion, we first propose that PHEV can induce atypical autophagy and jam the fusion of autophagosomes with lysosomes, thereby hampering the autophagy pathway degradation process. However, the specific mechanism of inhibiting autophagy maturation requires further exploration. On the other hand, such a seemingly contradictory process precisely sets the stage in the formation of early autophagy rather than the autophagolysosome. It may also be an evolutionary mechanism in which the virus escapes the immune system. Our studies provide insights into PHEV-host interactions and may develop a theoretical basis for further study between the virus and autophagy. These insights may raise promising targets for the development of antiviral strategies against PHEV infection.

## Author contributions

ND, WH designed the experiments, supervised the experiments, and analyzed the data. ND, KZ performed experiments and interpreted the data. YL, XL, ZL, JS, HL contributed reagents, materials, and analysis tools. ND, FG, WH drafted the article or revised it critically for important intellectual content. All authors agree with final approval of the version for submission.

### Conflict of interest statement

The authors declare that the research was conducted in the absence of any commercial or financial relationships that could be construed as a potential conflict of interest.

## References

[B1] Andries K. Pensaert M. (1981). Vomiting and wasting disease, a coronavirus infection of pigs. Adv. Exp. Med. Biol. 142, 399–408. 10.1007/978-1-4757-0456-3_33 6278894

[B2] Andries K. Pensaert M. B. (1980). Immunofluorescence studies on the pathogenesis of hemagglutinating encephalomyelitis virus infection in pigs after oronasal inoculation. Am. J. Vet. Res. 41, 1372–1378. 6255837

[B3] Beachboard D. C. Anderson-Daniels J. M. Denison M. R. (2015). Mutations across murine hepatitis virus nsp4 alter virus fitness and membrane modifications. J. Virol. 89, 2080–2089. 10.1128/JVI.02776-14 25473044 PMC4338892

[B4] Bjørkøy G. Lamark T. Brech A. Outzen H. Perander M. Overvatn A.. (2005). p62/SQSTM1 forms protein aggregates degraded by autophagy and has a protective effect on huntingtin-induced cell death. J. Cell Biol. 171, 603–614. 10.1083/jcb.200507002 16286508 PMC2171557

[B5] Blanchard E. Roingeard P. (2015). Virus-induced double-membrane vesicles. Cell. Microbiol. 17, 45–50. 10.1111/cmi.12372 25287059 PMC5640787

[B6] Burkard C. Verheije M. H. Wicht O. van Kasteren S. I. van Kuppeveld F. J. Haagmans B. L.. (2014). Coronavirus cell entry occurs through the endo-/lysosomal pathway in a proteolysis-dependent manner. PLoS Pathog. 10:e1004502. 10.1371/journal.ppat.1004502 25375324 PMC4223067

[B7] Chen X. J. Wang K. Xing Y. L. Tu J. Yang X. Zhao Q.. (2014). Coronavirus membrane-associated papain-like proteases induce autophagy through interacting with Beclin1 to negatively regulate antiviral innate immunity. Protein Cell 5, 912–927. 10.1007/s13238-014-0104-6 25311841 PMC4259884

[B8] Crawford S. E. Hyser J. M. Utama B. Estes M. K. (2012). Autophagy hijacked through viroporin-activated calcium/calmodulin-dependent kinase kinase-beta signaling is required for rotavirus replication. Proc. Natl. Acad. Sci. U.S.A. 109, E3405–E3413. 10.1073/pnas.1216539109 23184977 PMC3528557

[B9] Dong B. Gao W. Lu H. Zhao K. Ding N. Liu W.. (2015). A small region of porcine hemagglutinating encephalomyelitis virus spike protein interacts with the neural cell adhesion molecule. Intervirology 58, 130–137. 10.1159/000381060 25925196 PMC7179542

[B10] Dreux M. Chisari F. V. (2010). Viruses and the autophagy machinery. Cell Cycle 9, 1295–1307. 10.4161/cc.9.7.11109 20305376

[B11] Gannagé M. Dormann D. Albrecht R. Dengjel J. Torossi T. Rämer P. C.. (2009). Matrix protein 2 of influenza a virus blocks autophagosome fusion with lysosomes. Cell Host Microbe 6, 367–380. 10.1016/j.chom.2009.09.005 19837376 PMC2774833

[B12] Gao W. He W. Zhao K. Lu H. Ren W. Du C.. (2010). Identification of NCAM that interacts with the PHE-CoV spike protein. Virol. J. 7:254. 10.1186/1743-422X-7-254 20863409 PMC2955716

[B13] Hu B. Zhang Y. Jia L. Wu H. Fan C. Sun Y.. (2015). Binding of the pathogen receptor HSP90AA1 to avibirnavirus VP2 induces autophagy by inactivating the AKT-MTOR pathway. Autophagy 11, 503–515. 10.1080/15548627.2015.1017184 25714412 PMC4502722

[B14] Jackson W. T. Giddings T. H. Jr. Taylor M. P. Mulinyawe S. Rabinovitch M. Kopito R. R.. (2005). Subversion of cellular autophagosomal machinery by RNA viruses. PLoS Biol. 3:e156. 10.1371/journal.pbio.0030156 15884975 PMC1084330

[B15] Kimura S. Noda T. Yoshimori T. (2007). Dissection of the autophagosome maturation process by a novel reporter protein, tandem fluorescent-tagged LC3. Autophagy 3, 452–460. 10.4161/auto.4451 17534139

[B16] Klionsky D. J. Abdelmohsen K. Abe A. Abedin M. J. Abeliovich H. Acevedo Arozena A.. (2016). Guidelines for the use and interpretation of assays for monitoring autophagy (3rd edition). Autophagy 12, 1–222. 10.1080/15548627.2015.1100356 26799652 PMC4835977

[B17] Kroemer G. Mariño G. Levine B. (2010). Autophagy and the integrated stress response. Mol. Cell 40, 280–293. 10.1016/j.molcel.2010.09.023 20965422 PMC3127250

[B18] Lee Y. R. Lei H. Y. Liu M. T. Wang J. R. Chen S. H. Jiang-Shieh Y. F.. (2008). Autophagic machinery activated by dengue virus enhances virus replication. Virology 374, 240–248. 10.1016/j.virol.2008.02.016 18353420 PMC7103294

[B19] Levine B. Kroemer G. (2008). Autophagy in the pathogenesis of disease. Cell 132, 27–42. 10.1016/j.cell.2007.12.018 18191218 PMC2696814

[B20] Levine B. Mizushima N. Virgin H. W. (2011). Autophagy in immunity and inflammation. Nature 469, 323–335. 10.1038/nature09782 21248839 PMC3131688

[B21] Li Y. C. Bai W. Z. Hirano N. Hayashida T. Taniguchi T. Sugita Y.. (2013). Neurotropic virus tracing suggests a membranous-coating-mediated mechanism for transsynaptic communication. J. Comp. Neurol. 521, 203–212. 10.1002/cne.23171 22700307 PMC7162419

[B22] Li Z. He W. Lan Y. Zhao K. Lv X. Lu H.. (2016). The evidence of porcine hemagglutinating encephalomyelitis virus induced nonsuppurative encephalitis as the cause of death in piglets. PeerJ 4:e2443. 10.7717/peerj.2443 27672502 PMC5028786

[B23] Liu Y. Schiff M. Czymmek K. Tallóczy Z. Levine B. Dinesh-Kumar S. P. (2005). Autophagy regulates programmed cell death during the plant innate immune response. Cell 121, 567–577. 10.1016/j.cell.2005.03.007 15907470

[B24] Lussignol M. Queval C. Bernet-Camard M. F. Cotte-Laffitte J. Beau I. Codogno P.. (2013). The herpes simplex virus 1 Us11 protein inhibits autophagy through its interaction with the protein kinase PKR. J. Virol. 87, 859–871. 10.1128/JVI.01158-12 23115300 PMC3554085

[B25] Miller S. Krijnse-Locker J. (2008). Modification of intracellular membrane structures for virus replication. Nat. Rev. Microbiol. 6, 363–374. 10.1038/nrmicro1890 18414501 PMC7096853

[B26] Pankiv S. Clausen T. H. Lamark T. Brech A. Bruun J. A. Outzen H.. (2007). p62/SQSTM1 binds directly to Atg8/LC3 to facilitate degradation of ubiquitinated protein aggregates by autophagy. J. Biol. Chem. 282, 24131–24145. 10.1074/jbc.M702824200 17580304

[B27] Pei J. Zhao M. Ye Z. Gou H. Wang J. Yi L.. (2014). Autophagy enhances the replication of classical swine fever virus *in vitro*. Autophagy 10, 93–110. 10.4161/auto.26843 24262968 PMC4389882

[B28] Peng J. Zhu S. Hu L. Ye P. Wang Y. Tian Q.. (2016). Wild-type rabies virus induces autophagy in human and mouse neuroblastoma cell lines. Autophagy 12, 1704–1720. 10.1080/15548627.2016.1196315 27463027 PMC5079669

[B29] Petiot A. Ogier-Denis E. Blommaart E. F. Meijer A. J. Codogno P. (2000). Distinct classes of phosphatidylinositol 3′-kinases are involved in signaling pathways that control macroautophagy in HT-29 cells. J. Biol. Chem. 275, 992–998. 10.1074/jbc.275.2.992 10625637

[B30] Reggiori F. Monastyrska I. Verheije M. H. Calì T. Ulasli M. Bianchi S.. (2010). Coronaviruses hijack the LC3-I-positive EDEMosomes, ER-derived vesicles exporting short-lived ERAD regulators, for replication. Cell Host Microbe 7, 500–508. 10.1016/j.chom.2010.05.013 20542253 PMC7103375

[B31] Risco C. Sanmartín-Conesa E. Tzeng W. P. Frey T. K. Seybold V. de Groot R. J. (2012). Specific, sensitive, high-resolution detection of protein molecules in eukaryotic cells using metal-tagging transmission electron microscopy. Structure 20, 759–766. 10.1016/j.str.2012.04.001 22579245 PMC3351761

[B32] Rubinsztein D. C. Mariño G. Kroemer G. (2011). Autophagy and aging. Cell 146, 682–695. 10.1016/j.cell.2011.07.030 21884931

[B33] Shao S. Li S. Qin Y. Wang X. Yang Y. Bai H.. (2014). Spautin-1, a novel autophagy inhibitor, enhances imatinib-induced apoptosis in chronic myeloid leukemia. Int. J. Oncol. 44, 1661–1668. 10.3892/ijo.2014.2313 24585095 PMC6904104

[B34] Shintani T. Klionsky D. J. (2004). Autophagy in health and disease: a double-edged sword. Science 306, 990–995. 10.1126/science.1099993 15528435 PMC1705980

[B35] Suhy D. A. Giddings T. H. Jr. Kirkegaard K. (2000). Remodeling the endoplasmic reticulum by poliovirus infection and by individual viral proteins: an autophagy-like origin for virus-induced vesicles. J. Virol. 74, 8953–8965. 10.1128/JVI.74.19.8953-8965.2000 10982339 PMC102091

[B36] Xilouri M. Stefanis L. (2010). Autophagy in the central nervous system: implications for neurodegenerative disorders. CNS Neurol. Disord. Drug Targets 9, 701–719. 10.2174/187152710793237421 20942791

